# Acute Lung Injury- ARDS in H1N1: Timing of Therapy

**DOI:** 10.1186/1749-8090-10-S1-A134

**Published:** 2015-12-16

**Authors:** Ertay Boran, Mertay Boran, Pelin Çetin, Zahide Gümüş, Merve Andaç, Ali Nihat Annakkaya, Onur Ozlu

**Affiliations:** 1 Department of Anaesthesiology and Reanimation, Duzce University Faculty of Medicine, Duzce, Turkey; 2 Department of Thoracic Surgery, Duzce University Faculty of Medicine, Duzce, Turkey; 3 Department of Respiratory Disease, Duzce University Faculty of Medicine, Duzce, Turkey

## Background/Introduction

In some viral or bacterial infections, a minority of patients developed rapidly progressive pneumonia leading to acute lung injury (ALI)-acute respiratory distress syndrome (ARDS).

## Aims/Objectives

We reported two case of acute lung injury and their treatments.

## Method

34 year old male patient with severe acute respiratory failure was admitted to ICU. The patient was hospitalised with progressively worsened fever, coughing and dyspnoea lasting for one week. Severe pneumonia was first considered and antibiotics were started (levofloxacin, vancomycin) empirically and O2 was given through nasal canula. The clinic progressed to severe dyspnea in hours and after short Non-invasive ventilatory (NIV) support patient was entubated and accepted to ICU with severe ARDS. Oseltamavir 75 mg × 2 and Puls streoid therapy (1 mg/day) was added to antibiotics and patient mechanically ventilated. With no reply to the therapy the patient was scheduled to ECMO therapy and transferred to different ICU center where the same medical therapy continued under ECMO support. After a few weeks therapy patient clinics improved. The second case was 33 female patient with same clinic. After symptoms of fever, coughing for a week she was accepted to our ICU with ALİ-ARDS. The therapy of oseltamavir, vancomycin, levofloxasin, puls steroid (1 gr/day) and NIV was started. In a few days the patient's clinic improved. The culture results showed H1N1 infection.

## Results

Initial therapy of oseltamavir, pulse steroid therapy and NİV support results as suitable therapy for H1N1 induced ALI-ARDS. ECMO support is vital therapy in severe cases.

## Discussion/Conclusion

Suspicion of viral infections and timing of current therapy in H1N1 induced ALI-ARDS is a challenge for treatment.

## Consent

Written informed consent was obtained from the patient for publication of this abstract and any accompanying images. A copy of the written consent is available for review by the Editor of this journal

